# A community-based peer support service for persons with severe mental illness in China

**DOI:** 10.1186/s12888-018-1763-2

**Published:** 2018-06-04

**Authors:** Yunge Fan, Ning Ma, Liang Ma, Wei Xu, J. Steven Lamberti, Eric D. Caine

**Affiliations:** 1 Peking University Sixth Hospital; Peking University Institute of Mental Health; Key Laboratory of Mental Health, Ministry of Health (Peking University); National Clinical Research Center for Mental Disorders (Peking University Sixth Hospital), Beijing, China; 2 Beijing Chaoyang District Mental Disease Prevention and Control Center, The Third Hospital of Chaoyang District, Beijing, China; 3 Department of Psychiatry, University of Rochester Medical Center, Rochester, USA

**Keywords:** Severe mental illness, Peer support, Community, China

## Abstract

**Background:**

Peer support services for patients with severe mental illness (SMI) originated from Western countries and have become increasingly popular during the past twenty years. The aim of this paper is to describe a peer service model and its implementation in China, including the model’s feasibility and sustainability.

**Methods:**

A peer support service was developed in four Chinese communities. Implementation, feasibility and sustainability were assessed across five domains: Service process, service contents, peer training and supervision, service satisfaction, and service perceived benefit.

**Results:**

*Service process*: 214 peer support activities were held between July 2013 and June 2016. No adverse events occurred during three years. Each activity ranged from 40 to 120 min; most were conducted in a community rehabilitation center or community health care center. *Service content*: Activities focused on eight primary topics—daily life skills, social skills, knowledge of mental disorders, entertainment, fine motor skill practice, personal perceptions, healthy life style support, emotional support. *Peer training and supervision*: Intensive training was provided for all peers before they started to provide services. Regular supervision and continued training were provided thereafter; online supervision supplemented face to face meetings. *Service satisfaction*: Nineteen consumers (79.2%) (χ^2^(1) = 12.76, *p* < 0.001) were satisfied with the peers and 17 consumers (70.8%) (χ^2^(1) = 8.05, *p* = 0.005) expressed a strong desire to continue to participate in the service. Fourteen caregivers (93.3%) (χ^2^(1) = 11.27, *p* = 0.001) wanted the patients to continue to organize or participate in the service. *Service perceived benefit:* Six peers (85.7%) (χ^2^(1) = 3.57, *p* = 0.059) reported an improvement of working skills. Ten consumers (41.7%) (χ^2^(1) = 0.05, *p* = 0.827) reported better social communication skills. Six caregivers (40%) (χ^2^(1) = 1.67, *p* = 0.197) observed patients’ increase in social communication skills, five (33.3%) (χ^2^(1) = 1.67, *p* = 0.197) found their own mood had been improved.

**Conclusions:**

Peer support services for patients with SMI can be sustainably implemented within Chinese communities without adverse events that jeopardize safety and patient stability. Suggestions for future service development include having professionals give increased levels of support to peers at the beginning of a new program. A culturally consistent peer service manual, including peer role definition, peer training curriculum, and supervision methods, should be developed to help implement the service smoothly.

## Background

Peer support services are designed to bring together people with similar life experiences, culture, living environments, social status, concerns, and daily challenges [1]. This commonality promotes mutual respect, and it enables sharing of information, practical strategies and ongoing support that are critical to sustained behavior change [1]. Within the field of mental health services, peer service providers are individuals who have lived experience with mental illness, and who are willing to provide modeling and support to other individuals with mental illness [2]. Now regarded as a hallmark of recovery-oriented care [3], peer support services emphasize the importance of personal interests and strengths as foundations of recovery, rather than psychopathology and treatment considerations [4].

The benefits of peer support service are multifaceted, including benefits for peers (providers of peer support), consumers (receivers of peer support), the healthcare system, and society as a whole [5, 6]. Peers’ benefits include self-efficacy resulting from the experience of helping others [7], and earning money [8]. In addition, providing peer support services can promote peers’ communication skills, improve emotional and verbal expression, and increase social functioning [9]. All of these benefits contribute directly to the recovery process. The effects upon consumers with severe mental illness (SMI) are broad and include not only clinical benefits, such as fewer hospitalizations or psychiatric symptoms, but also personal and emotional benefits such as feelings of understanding, respect and trust [10, 11]. Peer support can also improve consumers’ social functioning and quality of life [12]. Peer support services are able to benefit healthcare systems and society as a whole. Peers, as healthcare service providers, can enrich mental health service teams while providing additional support to clinicians. In addition, since peers communicate with patients more often, they can function as important bridges between doctors and patients [13]. Peer support can also reduce social discrimination against persons with SMI. By demonstrating that people with SMI can recover and work, peer service providers are able to promote social understanding and acceptance, decrease social stigma and fear, and decrease patients’ feelings of shame and isolation [14].

Many studies have reported the importance of peers as positive role models of recovery and hope for consumers, caregivers and professional staff members alike [9, 15, 16]. Consumers as well as their caregivers become more positive and confident about themselves and their future, while peers can also help consumers like themselves better, believe in their own potential more and achieve their goals [9]. With regard to professional staff, they are able to see things differently and use other approaches to clients in specific situations [15].

The World Health Organization (WHO) Consultation documents the promise of peer support as an effective approach to chronic disease management and health promotion [17]. However, there is no widely accepted or standardized model for the operation of peer support services [18]. For example, peer support services in Rochester, NY, USA, are usually provided for a long-term duration and their contents are flexible. However, in New Zealand, peer run recovery houses are widely developed. The recovery house is an alternative to inpatient care in an acute psychiatric unit. It provides 24-h support, supervision and treatment [19]. In Australia, there is a Certificate IV Mental Health Peer Work as the minimum entry requirement for employment in peer support services, and their training content is relatively fixed [19, 20].

As for the format of peer service among patients with SMI, Davidson summarized it into three types: informal (naturally occurring) peer support, peer-run programs, and the employment of service recipients as service providers within traditional mental health programs [21]. Service duration can be long or short, and services can be located in the community or in hospitals. The content is varied, including activities such as disease and health education, social communication and daily life skills, and vocational support. Peer positions are often voluntary, and peers are typically selected by professionals based upon their communication skills, understanding of mental illness, personal responsibility and compassion, and level of clinical stability. In addition, professionals generally supervise peers during the delivery of support services [22]. Peer services currently operate in several countries including the United States [23], the United Kingdom [24], Australia [25], Germany [4], Brazil [26], New Zealand, and Canada [27]. The experiences of these countries have also proved that the operation of peer services should be consistent with local customs, values, and resource availability [28].

China, with its population of 1.3 billion, has more than 13 million persons suffering from psychotic disorders (hereafter labeled serious mental illness; SMI), based on an estimated prevalence of mood disorders was 6.1%, anxiety disorders was 5.6%, substance abuse disorders was 5.9%, and psychotic disorders was 1.0% [29]. At the end of 2014, there were only 25,307 psychiatrists (1.85/100,000 population) and 51,571 psychiatric nurses (3.77 /100,000 population) [30], very few psychotherapists and social workers, and almost no mental health occupational therapist. Outpatient mental health services for persons with SMI are offered primarily in large psychiatric hospital clinics, where one psychiatrist often sees 50–100 patients per day, with pharmacotherapy being the primary mode of treatment. If peers joined the mental health workforce and assisted in providing rehabilitation services, this strategy could enrich the scope of service and be beneficial to maintain patients’ good condition. It could also compensate for the lack of professional personnel in the long run.

This project was started in 2013 with the aim of establishing a community-based peer support service model for patients with SMI. No peer-based services existed in China at the time when we began the project. The project had the goal of demonstrating feasibility and sustainability, while also assessing whether the program would be associated with adverse patient events. In this paper, we described the implementation of a peer support service in China.

## Methods

### Participants

A peer support service was developed in four communities located in the Chaoyang district of Beijing: Tuanjiehu, Maizidian, Xiangheyuan, and Jinsong. Peer support was initiated in July 2013 in Tuanjiehu and Maizidian, and in the other two communities in August 2015.

All peer service providers were recommended by community doctors and evaluated by a research team psychiatrist. Peers who agreed to participate in were subsequently utilized if they met the following inclusion criteria: Diagnosed with schizophrenia or bipolar disorder which was recoded from their medical record provided by community doctors; age between 18 and 60 years old; stable at least 6 months, being adherent with medications according to patients’ and family members’ report, and having insight about their disease which was assessed through individual interviews conducted by psychiatrists; no drug or alcohol abuse; no severe medical illness; and having good social functioning which was assessed by the personal and social performance scale (PSP), more than 50 scores in PSP is required [31]. All participating peers were expected to be compassionate and willing to help others. Applicants with strong practical skills (e.g., cooking, drawing) were preferred.

All peers participated in intensive pre-service training. Once they finished training, posters were placed in diverse community locations to recruit patient-recipients (i.e., consumers); others came to the program based on the recommendation of community doctors. Each potential consumer was evaluated by the team psychiatrist, using the following inclusion criteria: diagnosed with schizophrenia or bipolar disorder; age 18–60 years old; stable at least 3 months; no drug or alcohol abuse; and no severe medical illness.

We recruited 12 peer providers and 50 consumers by 2016 (see Table 1). One peer in Tuanjiehu community quit in 2015, expressing concerns about too much pressure working as a peer. The caregivers of peers and consumers were also recruited if they were willing to be contacted for the follow-up evaluation. Fifteen caregivers (seven of peers, eight of consumers) from 15 families had been recruited in this study. The project proposal was reviewed and approved by Peking University Sixth Hospital Ethics Committee, and all peers and consumers provided written informed consent.

**Table 1:** Numbers of peers and consumers enrolled in each community by year

Role in each Community	Year				χ^2^ test of difference
	2013	2014	2015	2016	
Peer					χ^2^(9) = 10.30, *p* = 0.326
Tuanjiehu	5	5	4	4	
Maizidian	2	2	2	2	
Xiangheyuan	–	–	4	4	
Jingsong	–	–	2	2	
Total	7	7	12	12	
Consumer					χ^2^(9) = 27.73, *p* = 0.001
Tuanjiehu	10	11	14	14	
Maizidian	13	13	14	15	
Xiangheyuan	–	–	10	11	
Jingsong	–	–	10	10	
Total	23	24	48	50	

### Procedure

After the recruitment of peers and consumers, pre-service interviews with peers, consumers and their caregivers were conducted by a research assistant. The pre-service interviews were aimed to find out consumers’ needs in order to make our service contents meet their demands. As long as peers have received the intensive pre-service training, they were able to provide service. Apart from the intensive pre-service training that each peer was required to attend, peers were supervised during the whole service course. During the first year of the service, community doctors and clinical psychologists provided direction to peers before and after each peer support activity, and the psychiatrist provided guidance and support to community doctors and clinical psychologists. Beginning in July 2014 (the second year), community doctors offered non-scheduled “as needed” supervision which mainly depended on peers’ needs, while the clinical psychologists and psychiatrist offered group supervision every six weeks. After they had accumulated a year of experience, peers were able to prepare and organize session and training activities themselves; their credibility as service providers was well-established among consumers. In addition, two social workers were involved in this program beginning in March 2016, and they provided assistance before and after activities upon requests by the peers. These social workers also participated in group supervision. Along with face to face supervision, we also provided online supervision. A WeChat group was built which included peers, community doctors, social workers, clinical psychologists and psychiatrists.

An evaluation had been conducted among the peers, consumers, and their caregivers from the first two communities in December 2014, the evaluation concluded the service satisfaction and effectiveness.

### Measures

We examined implementation and feasibility of the community-based peer support service model for patients with SMI in China from five aspects – service process, service content, peer training and supervision, service satisfaction, and service perceived benefit.

#### Service process [32]

Measures of service process included the number of activities, frequency, average session time, and format of each activity. Peers were required to fill in a self-compiled form called “Activity Process Form” each time after the service being provided. The form included the date, start and end time of each activity, as well as the format of each activity. The forms had to be well-preserved by each peer and submitted to the project manager every six weeks.

#### Service content [33]

Measures of service content included the activity topics and the proportion of each. These were also tested by “Activity Process Form” which contained the aim and theme of each activity as well as consumers’ feedback. It was also required to be filled in after each service and submitted every six weeks.

#### Peer training and supervision [9]

Multi-background supervisor group was constituted by community doctors and professionals including psychiatrists, clinical psychologists and social workers. The measures of peer training and supervision included the contents of issues identified which were summarized from the training schedule and textbooks, audio and literal supervision records.

#### Service satisfaction [9, 18]

Semi-structured face to face interviews were conducted by a research assistant to measure the service satisfaction from the perspectives of consumers and their caregivers. The consumers were asked if they were satisfied with the peers and if they were willing to continue to participate in the peer support service. The caregivers were asked if they wanted the patients to continue to organize (for peers’ caregivers) or participate (for consumers’ caregivers) in the service. All the questions were supposed to be answered with yes or no.

#### Service perceived benefit [9, 15]

Feedback on the perceived impact of providing or receiving service was collected through brief structured face to face interviews conducted by a research assistant with peers, consumers as well as their caregivers. The peers and consumers were asked if organizing and participating in the service had any benefit to their improvement of skills or mood. The caregivers were also asked if the peer support service could improve their own mood or quality of life. All the questions were supposed to be answered with yes or no.

### Data analysis

All the forms as well as audio and literal records were recoded by three graduate students independently. Descriptive and inferential statistics analyses were used. Continuous variables were reported as means ± SD and were tested for normality with the Shapiro-Wilk test. Mean differences were tested by independent samples t-tests. Discrete variables were reported as N’s and percentages. Chi-square tests were used to examine differences. Analyses were performed with SPSS 19.0 for Windows.

## Results

### Peer and consumer participants

The numbers of peers and consumers enrolled in each community by year are shown in Table 1. There was a significant increase in consumers while non-significant in peers. Demographic information pertaining to peers and consumers is shown in Table 2. The mean ages of peers and consumers were 40.2 and 48.3, respectively. All peers and consumers were patients with SMI, most of them were diagnosed with schizophrenia. There was no significant difference between peers and consumers apart from age, working status and diagnosis. Peers were younger, less likely to be unemployed than consumers, and less peers had diagnoses of schizophrenia.

**Table 2:** The demographic information of consumers and peers

Characteristic	Peers		Consumers		*t* test or χ^2^ test of difference
	*N*	%	*N*	%	
Age (M ± SD)	40.2 ± 8.9		48.3 ± 8.2		*t* = −2.99, *p* = 0.004
Sex					χ^2^(1) = 2.37, *p* = 0.124
Male	8	66.7	21	42.0	
Female	4	33.3	29	58.0	
Education level					χ^2^(5) = 2.93, *p* = 0.711
Primary school	0	0	2	4.0	
Junior high school	1	8.3	10	20.0	
Senior high school	8	66.7	22	44.0	
Junior college	1	8.3	8	16.0	
Undergraduate degree	2	16.7	7	14.0	
Postgraduate degree	0	0	1	2.0	
Marital Status					χ^2^(3) = 1.32, *p* = 0.726
Single/never married	7	58.3	29	58.0	
Married	4	33.3	11	22.0	
Divorced	1	8.3	9	18.0	
Widowed	0	0	1	2.0	
Working status					χ^2^(1) = 5.53, *p* = 0.039
Yes (all part-time)	4	33.3	4	8.0	
No	8	66.7	46	92.0	
Diagnosis					χ^2^(1) = 10.61, *p* = 0.004
Schizophrenia	6	50.0	45	90.0	
Bipolar Disorder	6	50.0	5	10.0	

### Service process

**Fig. 1:**
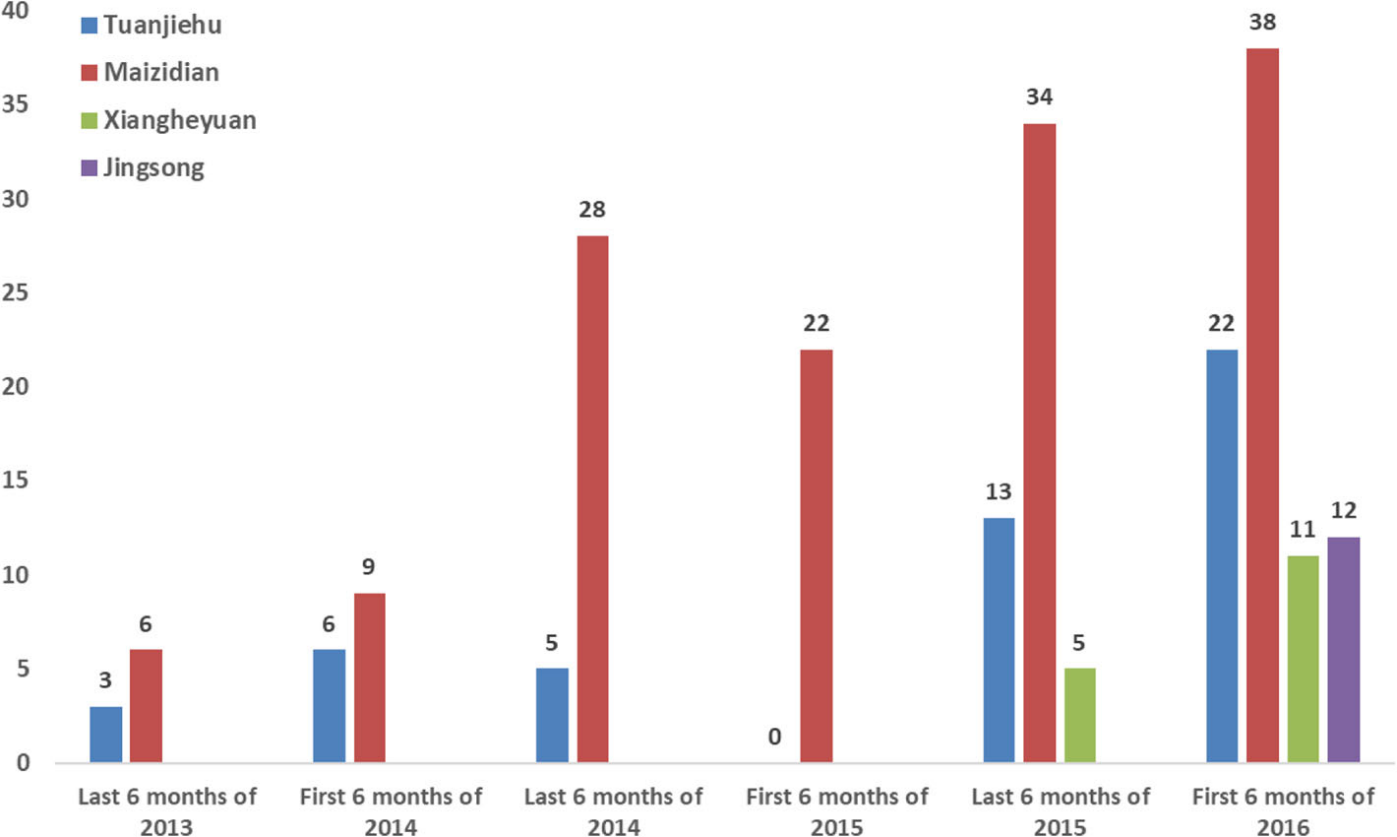
The number of peer support activities in each communities by every 6 months

A total of 214 peer support sessions were delivered from July 2013 to June 2016. One peer session means that peer met with consumers once and conduct activities or support service in groups. There was no report of violence, self-harm, or other potentially adverse events. The number of sessions for each community by every 6-month period is shown in Fig. 1. The numbers among different communities and time period yielded statistical significance (χ^2^(15) = 48.75, *p* < 0.001). During the first 6 months, only two communities developed peer support services and the peers were not yet proficient in running activities, which were organized about once every one or two months. During the second year, sessions were delivered about once every two weeks in these two communities. During the first 6 months of 2015, one peer in Tuanjiehu quit and one peer in Maizidian asked for a temporary leave because of needing to assist his sick mother. In addition, Tuanjiehu had trouble in finding the service place because the old one could not be used anymore. Resulting in no session had been provided during this time period in Tuanjiehu community. Beginning in August 2015, peer support services were started in another two communities. In 2015 and 2016, the session frequency differed among communities. It occurred once or twice each week in the first two communities, and once every two weeks in the newer two communities. The duration of each session ranged from 40 min to 120 min, depending on the activity theme and format for the session. The format of each activity varied depending on the topics, including group discussion, role play, personal sharing, lecture, and outdoor exercises. Most sessions were held in community rehabilitation centers or community health care centers. Each session typically involved at least two peers, with one leading and the other one assisting with service delivery, record keeping, and documenting consumers’ feedback.

### Service contents

On the basis of previous studies [34, 35], combining with the pre-service interviews, we summarized consumers’ needs into eight categories: daily life skills, social interpersonal skills, knowledge of mental disorders, entertainment, fine motor skill practice and exercise, personal perceptions such as self-esteem and self-confidence, healthy life style, and emotional support. The eight categories had been provided to peers before the initiation of peer support sessions. Peers had freedom to choose which category and what specific contents they were going to afford.

**Fig. 2:**
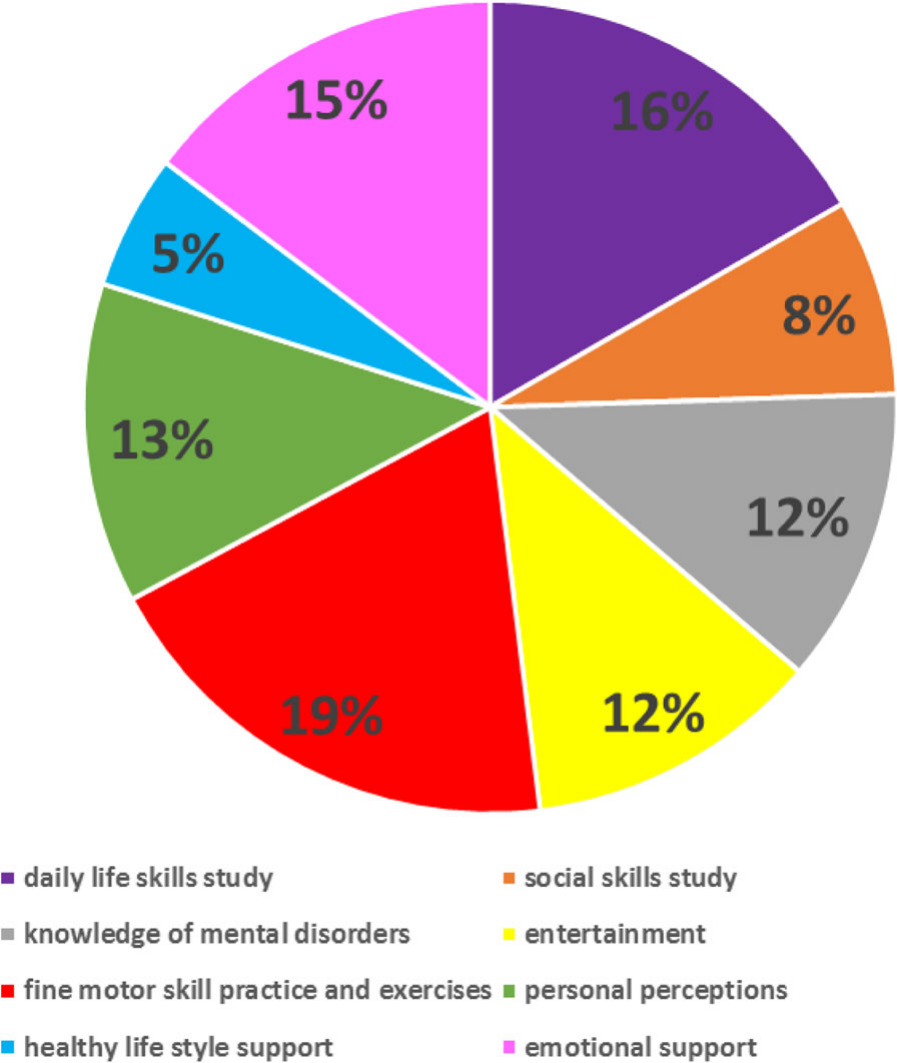
The proportion of peer support activity categories

Analyses of the service contents showed that each of the eight categories had been addressed during the three years’ service. Some of the session contents examples are listed below: daily life skills study (e.g., cooking, how to use WeChat), social skills study (e.g., communicating with body language and facial expression, verbal communication skills), knowledge of mental disorders (e.g., treatments for schizophrenia, recognizing disease recurrence, medications, side-effects), entertainment (e.g., singing, watching movies), fine motor skill practice and exercises (e.g., handwriting, puzzles, physical exercise), personal perceptions (building self-esteem and self-confidence), healthy life style support (e.g., controlling your emotions, healthy diets), emotional support (e.g., sharing stories of childhood, building relationships with family members). The proportion of each category is shown in Fig. 2. The top three most popular activity categories were fine motor skill practice and exercise (19%), daily life skills study (16%) and emotional support (15%).

The program began by peers sharing the rules of this service with those consumers who were referred or who responded to our posters. Each was asked to review and sign an informed consent document as well as a service and group activity agreement before beginning the activities.

In addition, there were usually three parts for each session. First, there were brief warm up activities, such as icebreaking games or setting-up exercises. The main activities relating to each topic then were initiated. At the end, peer providers or consumers would summarize the activity, followed by reciting a poem or singing a song together. Peers were required to complete documentation for each session. Before one session’s activities, they planned and completed an “Activity Plan Form”. Upon completion of each session, peers completed the “Activity Process Form” and a “Participant List”. If any adverse event or behavioral emergency (e.g., poorly controlled behavior, or problem that might require immediate attention) occurred during the session, peers were required to record the “Emergency Event Form” and to submit this report to the community doctors.

### Peer training and supervision

Pre-service training was provided for all peers. Training contents included the concept and theory of peer support services, working principles and requirements (including confidentiality, boundaries, and relationships), how to design and implement group activities, effective listening and speaking skills, how to handle emergency situations, and how to complete the record forms. We developed the training curriculum with reference of USA and Australia experience [8], and integrated with extensive discussion with potential peers during the pre-service interviews to better understand their knowledge and needs. The primary trainers were a psychiatrist and a clinical psychologist. Pre-service training was completed in five half a day with six workshop sessions. The format included coursework, group sharing and discussion, role play, and storytelling. We also included the peers from our first two communities when we trained new peers in June 2015 for the second two communities. The former shared their experiences and demonstrated how to conduct activities as part of their presentations.

With regard to the peer supervision during the whole service course, while the supervision group initially functioned to provide basic instruction and support related to conducting activities, the group subsequently focused more upon addressing peers’ difficulties and feelings of once they had accumulated service experience. For example, some peers might feel frustrated when they did not achieve their service goals, or when they encountered consumers who did not listen carefully or seemed bored. These experiences may undermine peers’ confidence, even though they are related to factors other than the peers’ level of skill or experience. The overall aim of supervision was to build peers’ confidence as well as their work capacity, motivation, and self-satisfaction.

In addition, face to face supervisions were supplemented with the online supervision. According to unstructured interviews with peers and community doctors, online group provided timely and efficient input to support both peer and service development. Whenever the peers experienced challenging work situations, they could ask questions in the online WeChat group. This forum provided an opportunity for the professional staff to respond quickly, and it also provided an opportunity for the other peers to express support and to give suggestions. The success of our online supervision prompted peers to build additional online peer support services. Now, three communities have their own WeChat groups for peers and consumers in order to provide timely support for consumers.

### Service satisfaction

Service satisfaction evaluation was only conducted among the first two communities (Tuanjiehu & Maizidian). Among the 24 consumers, three (12.5%) had not been assessed because of personal issues. Nineteen (79.2%) (χ^2^(1) = 12.76, *p* < 0.001) were satisfied with the peers and 17 (70.8%) (χ^2^(1) = 8.05, *p* = 0.005) expressed a strong desire to continue to participate in the service. Among the 15 caregivers, 14 (93.3%) (χ^2^(1) = 11.27, *p* = 0.001) wanted the patients to continue to organize or participate in the service.

### Service perceived benefit

Service perceived benefit was only assessed among the first two communities (Tuanjiehu & Maizidian). For peers, six (85.7%) (χ^2^(1) = 3.57, *p* = 0.059) reported an improvement of working skills, four (57.1%) (χ^2^(1) = 0.14, *p* = 0.705) reported increase in social communication skills. For consumers, ten (41.7%) (χ^2^(1) = 0.05, *p* = 0.827) reported better social communication skills. For caregivers, six (40%) (χ^2^(1) = 1.67, *p* = 0.197) observed patients’ increase of social communication skills, five (33.3%) (χ^2^(1) = 1.67, *p* = 0.197) found their own mood had been improved.

## Discussion

The results of this evaluation suggest that peer support services for persons with SMI are feasible and sustainable in China. The peer service has been in operation for three years, and the number of communities that developed peer support services has increased from two to four with a significant increase of consumers. The service has received positive evaluation and satisfaction from consumers and caregivers of both peers and consumers. Our experience can serve as a point of reference for other areas of China where there is interest in developing peer support services within their respective communities. Although we did not establish a standardized model for the operation of peer support services, our work may provide a general framework upon which to build such services.

Several aspects of this framework are particularly important early in the course of service development and implementation. It is important to provide peers with extra help and support in figuring out the topic of activities and preparing for peer support sessions at the beginning of service delivery. It should be recognized that the role of peers is very new in China, the majority of them are lacking in experience and confidence. At the beginning of the project, both the caregivers and community workers were worried about the safety of service, they did not believe in peers. Even the peers did not believe in themselves and they tended to avoid telling stories about themselves or sharing their own life experiences because of the perceived stigma and the unfamiliar environment. Therefore, we guided peers to provide some entertainment or skill training activities at first. It is helpful to identify community workers, such as doctors, nurses and social workers, who are willing to guide peers in preparing activity materials, and in providing on-site support as peers assume their new roles. The early availability of supportive health professionals serves to alleviate stress that is commonly experienced by peers as they adopt the role of service provider. At the same time, it is important for community workers to give peers more autonomy as their proficiency increases. We came to understand, as well, that many community health workers, including physicians, frequently have scant knowledge about SMIs and their treatment, and about providing peer support services. Mental health professionals should provide more guidance for community health workers from the knowledge, skill training and supervision in helping peer providers. It should also be noted that the frequency of sessions was less during the first year in our first two communities. This slower initial pace allowed us to gradually develop the necessary procedures and practices to support peer service provision. Our pace increased during the third year period, especially as we opened a second set of services and as the peers’ overall experience increased. In addition, after several months, the intimacy between peers and consumers also increased, then both the peers and community health workers became more positive about peer service.

It is essential to clarify the role of “peer.” At the outset, peers reflected that they are patients too – that is, consumers – and they initially expressed doubt that other consumers would trust and listen to them. Community workers also commented that it was difficult for them to decide how closely they should work with the peers in providing support. Clear job descriptions and role clarification is necessary for both peers and community workers. We observed that the role of peer service providers can vary according to the characteristics and needs of consumers and the activities planned for each session. These roles can include being a professional assistant, an advocate, a supporter, and even a case manager. Despite this variability in roles, we found that their primary responsibility involved working as a role model and partner in recovery for other consumers. As such, peer providers should be prepared to provide emotional support, living and social skills development, education, sharing of experiences, and encouragement.

We recommend offering a range of service topics as part of the peer training curriculum. In this project, peers initially found it difficult to generate their own ideas for group activities, preferring that professionals would provide a list of topics. In the absence of such guidance from professionals, there is a risk that peer providers could become somewhat overwhelmed and demoralized. To minimize the risk, we recommend that peers first consider carrying out activities that relate to topics with which they are familiar. These can include daily life skills (e.g., cooking), basic social skills (e.g., introducing oneself), information about mental illness (e.g., early warning signs of relapse), entertainment suggestions (e.g., watching movies), and fine motor skill practice and exercises (e.g., Chinese calligraphy).

It is important to provide specific training and continuing supervision for peers through the use of multidisciplinary groups, including psychiatrists, psychological therapists, community workers (especially primary care doctors), and social workers, depending upon their local availability. Pre-service training is particularly important for peers and community doctors in order to clearly understand their roles and job requirements, and also for obtaining the necessary skills for providing services. The content of our pre-service training turned out to be consistent with the experience in Hong Kong, which proved to be practical in non-Western context [36]. One of our peer program’s notable features is that we developed a group to provide supervision, an unusual feature in China. Several community doctors and peers mentioned that the supervision group effectively provided support for them. As for the format of supervision, we recommend combining both face-to-face and online formats. Face-to-face supervision is particularly helpful for affective interactions, while online supervision is more timely and efficient.

Finally, we recommend having at least two peers for each community, and to have both present at every session when possible. This level of peer staffing is particularly important for new programs. This team approach will better enable peers to monitor activities, and to adjust the peer role more effectively. Peer partners can support each other in a timely way before and after each session, discussing the plans and processes, sharing the job experiences, and identifying opportunities for improvement in future support and training activities. We received consistently positive feedback from peers and community doctors alike for having two peers working together by design.

There are some limitations in this study that need to be addressed. First, according to the results, although the service received positive evaluation and satisfaction from consumers and caregivers, the perceived benefit still not significant. This may because some of the effect have not been showed up by the evaluation or due to the small sample size, future researches are needed to replicate our study with larger sample size of peers, consumers as well as their caregivers. Secondly, based on our experience, at the initiate of peer service, peers tended to avoid telling stories about themselves or sharing their own life experiences because of the perceived stigma and the unfamiliar environment. However, the key idea of peer support service is that peer staff are trained to provide different services for which they are especially well suited based on their own life experiences and accumulated knowledge of how to live with a mental illness. So, although peers would adapt to their new roles eventually, future research could find more effective ways to speed up this process. We have also conducted follow-up evaluations with quantitative combined with qualitative measures among peers, consumers as well as their caregivers in the four communities, the results will be showed and discussed in another paper. Future research could also explore more long-term outcomes of peer support service in China.

## Conclusions

Peer support service for patients with SMI can be sustainably implemented within Chinese communities without adverse events that jeopardize safety and patient stability. Suggestions for future service development include having professionals give increased levels of support to peer service providers at the beginning of a new program to minimize peers’ anxiety and to promote rapid service model development. A culturally consistent peer support service manual, including information about peer role definition, the peer training curriculum, and supervision methods, should be developed to help peers implement the service smoothly.

## Abbreviation

SMI: severe mental illness
